# Demographic and Lifestyle Predictors of Prehypertension: A Cross-Sectional Study among Apparently Healthy Adults in Kumasi, Ghana

**DOI:** 10.1155/2019/1764079

**Published:** 2019-04-23

**Authors:** Eddie-Williams Owiredu, Ebenezer Dontoh, Selma E. S. Essuman, Bashiratu B. Bazanfara

**Affiliations:** ^1^Department of Medical Laboratory Technology, Faculty of Allied Health Sciences, Kwame Nkrumah University of Science and Technology, Kumasi, Ghana; ^2^Department of Nursing, Faculty of Allied Health Sciences, Kwame Nkrumah University of Science and Technology, Kumasi, Ghana; ^3^Department of Nursing, School of Applied Sciences, Central University, Accra, Ghana

## Abstract

**Background:**

Prehypertension has been shown to increase future risk of hypertension. Some demographic and lifestyle characteristics have been implicated to increase the risk of development of prehypertension. Yet, there is paucity of data on the current prevalence of prehypertension and its associated risk factors in Ghana. This study evaluated the prevalence of prehypertension and examined the demographic and lifestyle characteristics associated with prehypertension among apparently healthy Ghanaian adults in Kumasi.

**Methods:**

This was a cross-sectional study conducted from March to April, 2018, in Kumasi, Ghana. A total of 204 participants (80 males, 124 females, 25 years and above) who reported not diagnosed of hypertension and not on any antihypertensive medication were included in the study. Validated questionnaire was used to obtain sociodemographic and lifestyle characteristics of study participants. Height and weight of each respondent were measured and their corresponding Body Mass Index (BMI) was calculated. Blood pressure (BP) was measured with an automated blood pressure apparatus from the right arm. Prehypertension was defined as systolic BP of 120-139 mmHg and/or diastolic BP of 80-89 mmHg.

**Results:**

Out of 204 participants, the prevalence of prehypertension was 49.0%. From multivariate logistic regression models, having lower level of education [aOR=2.74, 95% CI (1.15-6.55),* p*=0.02], not practicing at least 30 min daily walks [aOR=2.59, 95% CI (1.31-5.10),* p*=0.01], not exercising routinely [aOR=1.93, 95% CI (0.97-3.85),* p*=0.04], and alcohol consumption [aOR=3.58(1.52-8.46), p=0.004] were independently associated with higher odds of prehypertension.

**Conclusion:**

The prevalence of prehypertension is high among apparently healthy Ghanaian adults (49.0%). Lower educational level, sedentary lifestyle, and alcohol consumption are the predominant risk factors for prehypertension in Kumasi.

## 1. Introduction

Cardiovascular disease (CVD) is emerging as a major public health burden in sub-Saharan Africa, with hypertension being a key risk factor [1]. Hypertension, a common health problem worldwide, is an important modifiable risk factor for CVD [2]. Prehypertension, an initial stage of hypertension, where preemptive measures have been shown to be effective in delaying or preventing the onset of the disease [3, 4], has been linked with increased future risk of hypertension as well as CVD [5].

In Ghana, hypertension is the second leading cause of outpatient morbidity among adults [6], with prevalence ranging from 19 to 48% [7, 8]. The high morbidity has been associated with inadequate rates of detection, treatment, and control [6] as well as noncompliance to medication and regular checkups [9].

Development of health policies for prevention and control of hypertension requires consistent evidence on the prevalence of hypertension in distinct regions [10]. However, the paucity of such reliable data on the prevalence of major cardiovascular risk factors in Ghana has been identified as a major obstacle to developing appropriate policies and interventions for CVD in the region [6]. Furthermore, report from the Framingham Heart study indicates that clinical hypertension in people with prehypertension is approximately 18-37% times in those aged 35–64 years and 26-50% times in those aged ≥ 65 years [5] compared to normotensives, indicating the imminence of an increase rate of clinical hypertension and consequently, higher prevalence of CVD mortality [11] if high risk subjects are not identified and prehypertension is controlled.

This study was, thus, undertaken to evaluate the prevalence of prehypertension and examine demographic and lifestyle characteristics/risk factors associated with prehypertension among apparently healthy Ghanaian adults in Kumasi, Ghana. The findings of this study would equip policy makers with the necessary information to develop apt strategies to abate the increasing prevalence of the disease and its associated risk factors as well as creating awareness among high risk population.

## 2. Materials and Methods

### 2.1. Study Design/Setting

This cross-sectional study was conducted from March to April, 2018, among apparently healthy participants living in Kumasi, Ghana. Kumasi is the second largest city in Ghana and the capital of the erstwhile medieval Ashanti Kingdom, Gold Coast. It is currently the headquarters of the Ashanti region and lies between latitude 6.35°N and 6.40°N and longitude 1.3°W and 1.35°W [12]. It is 150sq km in size and located in the rainforest zone of West Africa with a population of about 1,468,609 inhabitants [13].

### 2.2. Study Population

The sample size for this study was calculated using the MedCalc Statistical Software version 18.9.1 (MedCalc Software bvba, Ostend, Belgium). Based on the most recent prevalence of prehypertension reported in Kumasi (40.0%) [1], a 95% confidence level, response distribution of 50%, 5% margin of error, a study power of 80%, and design effect of 1, the minimum sample size required for this study was 194.

### 2.3. Participants' Recruitment

A total of 204 Ghanaian adults, 25 years and above, who reported not diagnosed of hypertension and not on any antihypertensive medication were recruited for this study. Included subjects were residents of Kumasi, Ghana. Kumasi was selected because it is the largest and one of the most populated areas in the Ashanti region of Ghana. Kumasi has approximately four hundred thousand households with an average household size of about four persons according to the 2010 Population and Housing Census [14]. This served as the sampling frame for the study. A two-stage simple random sampling technique was used for participant recruitment. Briefly, investigators first approached households randomly selected from the sampling frame. Secondly, out of the eligible individuals in each selected household, one person is randomly selected for face-to-face data collection. A household was defined as a group of people who usually live under the same roof and share meals. If more than one household was present in the same dwelling, one was randomly selected. Pregnant women and children were excluded. Elaborate pilot-tested questionnaire, designed by reviewing previous studies of similar objective and customized to fit our study objectives, was used to obtain sociodemographic and lifestyle characteristics of study participants in a language they could easily comprehend. Data collected include age, sex, highest educational level, marital status, employment status, physical activity, fruits and vegetable intake, alcohol consumption, and family history of hypertension.

### 2.4. Anthropometric and Blood Pressure Measurement

The weight of the respondents was measured in light clothing without shoes, in an upright position to using a calibrated analogue scale (Seca, Hamburg, Deutschland). Height was measured without shoes using a stadiometer (Seca, Hamburg, Deutschland). Body mass index (BMI) was calculated using the equation [BMI (kg/m^2^) = weight/height^2^].

Blood pressure (BP) was measured using the World Health Organization (WHO) protocol with an identical BP apparatus. Participants were asked to rest for at least five minutes before measurement. Blood pressure was measured with an automated blood pressure apparatus (Omron MX3-Omron Matsusaka Co., Ltd. Japan) from the right arm. The average of the two readings taken five minutes apart was recorded as the blood pressure measurement. All the instruments used in the study were calibrated prior to the commencement of the study.

### 2.5. Definition of Terms

Prehypertension was defined as systolic BP of 120-139 mmHg, and/or diastolic BP of 80-89 mmHg according to Joint National Committee 7 criteria [15]. Overweight was defined as BMI ≥ 25.00-29.99 kg/m^2^ according to the WHO standards [16]. Participants considered to be alcohol consumers were those who take in alcoholic beverages: at least one drink (e.g., beer, wine, spirits)/week. Alcohol consumption status was based on the items: “Have you ever consumed any alcoholic beverage?” (1), “Do you still have the habit or have you completely quit?” (2), “How often do you take in alcohol?” (3). Subjects who do not consume fruit/vegetables or consume fruit/vegetables but less than once per week were classified as having infrequent fruit & vegetables intake [17] based on the items: “How many days in the past week had fruits (1), and green vegetables (2) been taken?”, “How often do you consume fruits (3) and vegetables (4)?”. Subjects who had lower than secondary education as their highest attained educational level were considered as having a low level of education. In accordance with the WHO standards, routine exercise was defined as engaging in at least 150 minutes of moderate-intensity aerobic physical activity throughout the week or engaging in at least 75 minutes of vigorous-intensity aerobic physical activity throughout the week or an equivalent combination of moderate- and vigorous-intensity activity [18]. Routine exercise was based on the items: “Do you regularly exercise?” (1), “What kind of exercise do you engage in?” (2), “How long does it take when you are exercising?” (3).

### 2.6. Data Analysis

Chi-square and Fishers exact tests were used to compare categorical data and Independent t-test was used to compare continuous data where applicable. Multivariate logistic regression analysis was performed to identify factors associated with prehypertension. All the results from the logistic regression model are presented as odds ratios with corresponding 95% confidence intervals. All statistical tests were two-tailed and a* p* value < 0.05 was considered statistically significant. All statistical analyses were performed using IBM SPSS 25.0 Statistics.

### 2.7. Ethical Considerations

Ethical approval for this study was obtained from the Committee on Human Research Publication and Ethics (CHRPE) of the School of Medical Sciences, Kwame Nkrumah University of Science and Technology.

## 3. Results

Table 1 shows the sociodemographic and lifestyle characteristics of the study population. The mean age of the study population was 45.63 years. A higher proportion of the study participants were greater than 40 years old (54.9%), were females (60.8%), had secondary education or lower (86.3%), were married (64.7%), were employed (86.3), do not routinely exercise (78.4%), practice at least 30-minute daily walks (76.5%), frequently take in fruit and vegetables (74.0%), do not consume alcohol (84.8%), and 39.2% had family history of hypertension (Table 1).

The anthropometric and haemodynamic characteristics of the study population are shown in Table 2. The mean height, weight, BMI, systolic blood pressure, and diastolic blood pressure were 1.63 m, 71.49 kg, 27.07 kg/m^2^, 116.39 mmHg, and 77.31 mmHg, respectively. Majority of the study participants were overweight (60.8%) (Table 2).

Table 3 shows the sociodemographic and lifestyle characteristics of the study population stratified by hypertension status. The prevalence of prehypertension among the study population was 49.0%. Prehypertension was significantly associated with lower educational level (52.3%;* p* = 0.03), subjects who did not practice at least 30 minutes daily walks (66.7%;* p* = 0.01), and alcohol consumption (74.2%;* p* = 0.003) (Table 3).

In the multivariate logistic regression model, having lower level of education [aOR = 2.74, 95% CI (1.15-6.55), p= 0.02], not practicing at least 30 minutes daily walks [aOR = 2.59, 95% CI (1.31-5.10), p = 0.01], not exercising routinely [aOR = 1.93, 95% CI (0.97-3.85), p = 0.04], and alcohol consumption [aOR = 3.58, 95% CI (1.52-8.46), p= 0.004] were independently associated with higher odds of prehypertension (Table 4).

## 4. Discussion

Cardiovascular disease (CVD) is one of the major public health problems in sub-Saharan Africa, including Ghana, with 2 million deaths annually [19]. Hypertension has been identified as a key risk factor of CVD [1] and prehypertension has been linked with increased future risk of hypertension as well as CVD [5]. A study by Qureshi et al. found that subjects with prehypertension had a two-fold risk of developing clinical hypertension compared to normotensive subjects [20]. Furthermore, according to a longitudinal analyses of nonhypertensive subjects in the Framingham Heart study, the 4–year incidence of clinical hypertension in people with prehypertension is approximately 18-37% times in those aged 35–64 years and 26-50% times in those aged ≥ 65 years compared to normotensives [5]. This shows the imminence of an increase rate of clinical hypertension and consequently, higher prevalence of CVD mortality [11] if high risk subjects are not identified and prehypertension controlled. As such, study was undertaken to evaluate the prevalence of prehypertension and examine demographic and lifestyle characteristics/risk factors associated with prehypertension among apparently healthy Ghanaian adults in Kumasi, Ghana.

Previous studies have reported varying prevalence rates of prehypertension. A study by Agyemang and Owusu-Dabo reported a prevalence of 40.0% among adults in the Ashanti region of Ghana [1]. A study by Incoom et al. reported a prevalence of 25.4% among adults in the Hohoe Municipality of Ghana [21]. Similarly, Atinyi et al., in a study among adults in Keta Municipality of Ghana, reported a prevalence of 27.3% [22]. Another study by Bani et al. among traders in Hohoe Municipality, Ghana, reported a prevalence of 33.8% [23]. Also, in a meta-analysis by Gebreselassie and Padyab, the weighted prevalence of prehypertension in Ghana was 30.7% in 2014 [24]. Some international disparities have also been observed. In 2010, Mayo et al. revealed a prehypertension prevalence of 12.0% among individuals from Thailand [25] and Gebreselassie and Padyab revealed a prevalence of 29.4% among people from South Africa [24]. This present study reports a prevalence of 49.0% among apparently healthy participants in Kumasi, Ghana. The prevalence observed in this study is higher compared to previous studies in Ghana as well as other countries. The higher prevalence rate in this study may be attributed to the prevalence of diet-related health conditions which tend to increase with time due to nutritional transition and globalization as well as unhealthy lifestyle habits among the general Ghanaian populace [26].

The strength of this study is the identification of risk factors for prehypertension among apparently healthy population. A pilot study of a nutritional education program conducted by Cappuccio et al. in Ghana resulted in a reduction in the mean systolic and diastolic blood pressure by 6.4 mmHg and 4.5 mmHg, respectively, within four weeks [27]. Our finding that the likelihood of being prehypertensive increases with lower level of education [aOR = 2.74, 95% CI (1.15-6.55),* p *= 0.02] was, thus, expected. This finding is also consistent with a study by Bushara et al. in North Sudan [28] and Steyn et al. in South Africa [29], who reported an association between low educational level and risk of hypertension.

Another finding of this study is that sedentary lifestyle (not practicing at least 30 minutes daily walks [aOR = 2.59, 95% CI (1.31-5.10),* P *= 0.01] and not exercising routinely [aOR = 1.93, 95% CI (0.97-3.85),* P* = 0.04]) pose an increased risk of prehypertension among apparently healthy Ghanaian adults. This finding is in harmony with a study by Shukla et al. [19], who also reported that sedentary lifestyle among apparently healthy Western Indian population was associated with increased risk of prehypertension. This finding may be attributed to the likelihood of sedentary lifestyle to influence the development of obesity, which has been linked to the development of hypertension through diverse mechanisms including metabolic, endothelial and vascular dysfunction, neuroendocrine imbalances, sodium retention, glomerular hyperfiltration, proteinuria, and maladaptive immune and inflammatory responses [30].

This study also found alcohol consumption [aOR = 3.58, 95% CI (1.52-8.46),* P* = 0.004] to be significantly associated with higher risk of prehypertension, as consistent with a study by Steyn et al. in South Africa [29]. The mechanism by which alcohol predisposes to hypertension is still under investigation. However, proposed mechanisms include imbalance of the central nervous system, impairment of the baroreceptors, enhanced sympathetic activity, stimulation of the renin-angiotensin-aldosterone system, increased cortisol levels, increased vascular reactivity due to increase in intracellular calcium levels, stimulation of the endothelium to release vasoconstrictors, and loss of relaxation due to inflammation and oxidative injury of the endothelium leading to inhibition of endothelium-dependent nitric oxide production [31].

Early targeting of prehypertensive individuals and the adherence to lifestyle modifications may provide significant long-term benefits, especially in resource-poor settings where the control of hypertension is a serious problem. Education and promotion of physical activity and advocating for the avoidance/ reduction of alcohol consumption may have a profound positive effect in abating clinical hypertension in Ghana.

### 4.1. Strengths and Limitations

The major strength of this study is the identification of risk factors for prehypertension among apparently healthy adults in Kumasi, Ghana. Additionally, aside being regionally representative, this study has the advantage of providing accurate regional data as data quality monitoring was performed throughout the study period. On the contrary, as with most epidemiological studies, this study is limited by the fact that the blood pressure levels were based on the average of two measurements at a single visit, which might have overestimated the prevalence rates. Moreover, the cross-sectional nature of the study design precludes the establishment of the cause-effect relationship between sociodemographic and lifestyle predictors and prehypertension. Thus, follow-up studies of longer periods to assess the association are warranted. Additionally, the study population was skewed towards having lower than secondary education which may have resulted in the increased risk of prehypertension among subjects with lower educational level. Nonetheless, the data obtained provides a possible representation of the distribution of prehypertensives in Kumasi.

## 5. Conclusion

The prevalence of prehypertension is high among apparently healthy Ghanaians (49.0%). Major risk factors for the development of prehypertension in Kumasi are lower level of education, sedentary lifestyle, and alcohol use. Routine screening programs may help identify high risk individuals and public health education on risks factors of prehypertension, emphasizing the need for regular check-ups and educating the public on the risk of prehypertension transitioning to clinical hypertension may help abate the increasing prevalence of the disease.

## Figures and Tables

**Table 1:** Sociodemographic and lifestyle characteristics of the study population.

Variables	Mean ± SD
*Age (years)*	45.63 ± 13.36
	*n(%)*
<40 years	92(45.1)
≥40 years	112(54.9)
*Sex*	
Male	80(39.2)
Female	124(60.8)
*Educational level*	
≤Secondary	176(86.3)
>Secondary	28(13.7)
*Marital status*	
Single	72(35.3)
Married	132(64.7)
*Employment status*	
Unemployed	28(13.7)
Employed	176(86.3)
*Practice at least 30 min daily walks*	
Yes	156(76.5)
No	48(23.5)
*Routine exercise*	
Yes	44(21.6)
No	160(78.4)
*Frequent intake of fruits and vegetables*	
Yes	151(74.0)
No	53(26.0)
*Alcohol consumption*	
Yes	31(15.2)
No	173(84.8)
*Family history of hypertension*	
Yes	80(39.2)
No	124(60.8)

**Table 2:** Anthropometric and haemodynamic characteristics of the study population.

Variables	Mean ± SD
Height (m)	1.63 ± 0.09
Weight (kg)	71.49 ± 15.85
BMI (kg/m^2^)	27.07 ± 5.69
*Normal*	*80(39.2)*
*Overweight*	*124(60.8)*
SBP (mmHg)	116.39 ± 15.70
DBP (mmHg)	77.31 ± 9.28

BMI; Body Mass Index, Normal BMI; 18.5-24.9, Overweight; ≥ 25.00-29.99, SBP; Systolic Blood Pressure, DBP; Diastolic Blood Pressure.

**Table 3:** Sociodemographic and lifestyle characteristics of the study population stratified by hypertensive status.

Variables	Normotensive (n=104/51%)	Pre-HTN (n=100/49%)	*p* values
*Age (years)*	46.54 ± 15.59	44.68 ± 10.55	0.32
*Age ranges*			0.16
<40 years	52(56.5)	40(43.5)	
≥40 years	52(46.4)	60(53.6)	
*Sex*			0.20
Male	36(45.0)	44(55.0)	
Female	68(54.8)	56(45.2)	
*Highest educational level*			**0.03**
≤Secondary	84(47.7)	92(52.3)	
>Secondary	20(71.4)	8(28.6)	
*Marital status*			0.19
Single	32(44.4)	40(55.6)	
Married	72(54.5)	60(45.5)	
*Employment status*			0.42
Unemployed	12(42.9)	16(57.1)	
Employed	92(52.3)	84(47.7)	
*Practice at least 30 min daily walks*			**0.01**
Yes	88(56.4)	68(43.6)	
No	16(33.3)	32(66.7)	
*Routinely exercise*			0.06
Yes	28(63.6)	16(36.4)	
No	76(47.5)	84(53.5)	
*Frequent intake of fruit & vegetables*			0.21
Yes	81(53.6)	70(46.4)	
No	23(43.4)	30(56.6)	
*Alcohol consumption*			**0.003**
Yes	8(25.8)	23(74.2)	
No	96(55.5)	77(44.5)	
*Family history of hypertension*			0.39
Yes	44(55.0)	36(45.0)	
No	60(48.4)	64(51.6)	

Chi-square and Fisher exact test were performed to compare categorical variables. Independent t-test was used to compare continuous variables. *P* values of significant variables are in bold print. Pre-HTN; Prehypertension, defined by systolic BP of 120-139 mmHg, and/or diastolic BP of 80-89 mmHg.

**Table 4:** Multivariate logistic regression analyses of factors associated with prehypertension among the study population.

Variable	aOR (95% CI)	*p* value
Male	1.48(0.84-2.61)	0.17
≥40 years	1.50(0.86-2.61)	0.15
≤Secondary education	2.74(1.15-6.55)	**0.02**
Single	1.50(0.84-2.67)	0.17
Unemployed	1.46(.65-3.27)	0.36
Not practicing at least 30 minutes daily walks	2.59(1.31-5.10)	**0.01**
Not exercising routinely	1.93(0.97-3.85)	**0.04**
Infrequent intake of fruit & vegetables	1.51(0.80-2.84)	0.20
Alcohol consumption	3.58(1.52-8.46)	**0.004**
Overweight	1.29(0.74-2.24)	0.37
Family history of hypertension	1.30(0.74-2.29)	0.36

Multivariate logistic regression analysis was performed to determine independent predictors of prehypertension. *p* < 0.05 was considered statistically significant (*p* values of significant variables are in bold print).

## Data Availability

All relevant data are within the article.
